# Mechanistic insights into Na^+^ pumping by KR2: Distinct roles of Asp102 and Asn112 coupled with retinal distortion in two O intermediates

**DOI:** 10.1016/j.jbc.2026.111313

**Published:** 2026-02-26

**Authors:** Sahoko Tomida, Rei Abe-Yoshizumi, Akimori Wada, Hideki Kandori, Yuji Furutani

**Affiliations:** 1 Department of Life Science and Applied Chemistry, Nagoya Institute of Technology, Nagoya, Japan; 2 Laboratory of Organic Chemistry for Life Science, Kobe Pharmaceutical University, Kobe, Japan; 3 OptoBioTechnology Research Center, Nagoya Institute of Technology, Nagoya, Japan

**Keywords:** bioenergetics, biophysics, fourier transform IR (FTIR), photobiology, protein dynamic, rhodopsin, sodium transport, cation pump, time-resolved spectroscopy

## Abstract

*Krokinobacter eikastus* rhodopsin 2 (KR2) is a light-driven Na^+^ pump. In the initial state, a sodium ion does not bind near the protonated retinal Schiff base due to electrostatic repulsion and is instead taken up during formation of the O intermediate in the photocycle. Previous cryogenic and time-resolved crystallographic studies showed that Na^+^ can be coordinated by either Asn112 or Asp252 near the protonated retinal Schiff base. In addition, KR2 forms a pentamer in which each protomer binds a Na^+^ at Asp102 located at the interfacial region, although the functional relevance of this site has remained unclear. Here, we used time-resolved Fourier-transform infrared spectroscopy to clarify the Na^+^ translocation mechanism. Four states—K, L/M, and two O (O_1_ and O_2_) states—were spectrally resolved and characterized using site-directed mutants and isotopically labeled retinal analogs. C–C stretching vibrations indicated that O_1_ and O_2_ contain 13-*cis* and all-*trans* retinal configurations, respectively. The C=O stretching band of Asn112 was most intense in the O_1_ state, consistent with close Na^+^ interaction at this residue. Retinal hydrogen out-of-plane vibrational modes further revealed enhanced torsion around the C_11_, C_12_, and C_15_ positions specifically in O_1_. Importantly, the decay of the O_2_ intermediate was markedly slowed at high Na ^+^ concentration in the D102N mutant, suggesting that Na^+^ rebinding at Asp102 may facilitate Na^+^ release along the exit pathway during the O_2_ intermediate. These findings provide a unified mechanistic model in which coordinated retinal distortion and site-specific ion interactions drive directional Na^+^ pumping through the two O intermediates of KR2.

Microbial rhodopsins (type-1 rhodopsins) are widely distributed among diverse organisms (1). Their fundamental molecular architecture consists of seven transmembrane α-helices and a chromophore, all-*trans* retinal, covalently linked to a lysine residue on the seventh helix (TM7) *via* a protonated retinal Schiff base (PRSB) (1). Bacteriorhodopsin (BR), discovered in 1971 in *Halobacterium salinarum* (2), is the most extensively studied microbial rhodopsin. BR functions as a light-driven outward proton pump and forms two-dimensional crystals, the so-called purple membrane. Upon light activation, BR proceeds through a photocycle involving K, L, M, N, and O intermediates before returning to the dark state within several tens of milliseconds (3). Two aspartates (Asp85 and Asp96) and one threonine (Thr89) located in TM3 constitute the DTD motif which is critical for proton pumping. Time-resolved FTIR studies established that Asp85 and Asp96 act as the proton acceptor and donor for the PRSB, respectively, based on their characteristic protonation changes during M-state formation and decay (4, 5, 6). Three residues at the corresponding TM3 positions have thus been recognized as a functional signature for microbial rhodopsins. For example, outward proton pumps typically possess the DTD motif, whereas inward chloride pumps from *H*. *salinarum* and *Nonlabens marinus* contain the TSA and NTQ motifs, respectively (7, 8). Replacement of the Asp residue at the first motif position with Thr or Ser eliminates the counterion for the PRSB, enabling chloride to serve simultaneously as both counterion and transported substrate (9, 10). In both proton- and chloride-pumping rhodopsins, the substrate ion resides near the PRSB in the dark state; therefore, atomic-level features of the ion-binding site and its structural changes during transport have been intensively investigated (11, 12, 13, 14).

For many years after the discovery of BR, it was believed that microbial rhodopsins could not pump cations other than protons, due to the presence of the positively charged PRSB along the ion-transport pathway. This view was overturned by the discovery of the first light-driven sodium pump rhodopsin, KR2, from the marine flavobacterium *Krokinobacter eikastus* in 2013 (15). KR2 contains an NDQ motif in TM3, and its crystal structure revealed no Na^+^ bound near the PRSB (Fig. S1, *a* and *b*) (16). Analyses by AFM, crystallography, and FTIR have shown that KR2 assembles into a pentamer and that Na^+^ is bound at the extracellular interface between protomers (15, 16, 17, 18). Thus, light-driven sodium-pumping rhodopsins introduced a new concept of active transport, as they do not bind the transport substrate, the sodium ion, near the PRSB in the dark state (19).

Thus far, mechanistic proposals for Na^+^ transport by KR2 have largely relied on static crystal structures (16, 18, 20). The first model was based on pH-dependent reorientation of Asp116, the counterion of the PRSB. At acidic pH, the Asp116 side chain points toward Asn112 and Ser70 (Fig. S1*a*), whereas at neutral pH, it faces the PRSB (Fig. S1*b*). Kato *et al*. proposed that Asp116 flips away from the PRSB upon protonation in the M state, isolating the positive charge and permitting Na^+^ passage. After Na^+^ transits the Schiff base, Asp116 flips back to reprotonate the PRSB, thereby preventing backflow (Fig. S1, *a* and *b*) (16). In contrast, Kovalev *et al*. determined crystal structures of both the dark state (18) and the O intermediate (Fig. 1, *A* and *B*) (21). In the dark state, Na^+^ is coordinated at an extracellular site comprising Asp102 and residues Tyr25, Thr83, and Phe86 from an adjacent protomer (18).

**Figure 1:**
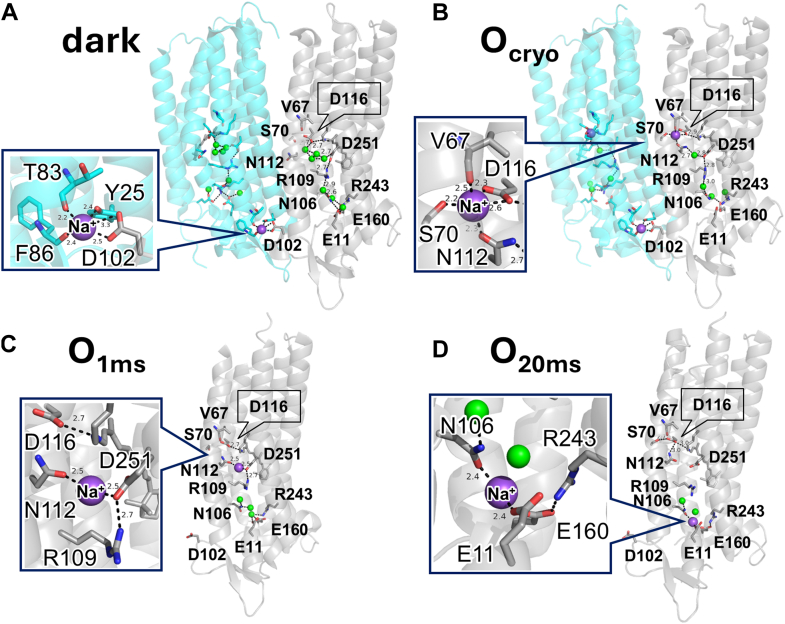
**X-ray crystallographic structures of *Krokinobacter* rhodopsin 2 (KR2)**. The structures enlarged around the Na ^+^-binding sites are shown in insets. The dark state and O intermediate of KR2 reported in Kovalev *et al*. (PDB ID: 6YC3 and 6XYT) are shown in (*A*) and (*B*), respectively (21). Two protomers are colored *cyan* and *gray*. The crystal structures recorded with 1 ms and 20 ms delay after laser excitation reported in Skopintsev *et al*. (PDB ID: 6TK2 and 6TK1) are shown in (*C*) and (*D*), respectively (22). The dark state structure (Fig. S1*C*) was almost same as those obtained by previous studies. All-*trans* retinal and side chains of key amino acid residues are shown by stick drawing. *Green* spheres represent water molecules. The numbers near *dotted lines* represent the distance (Å) between the atoms connected by each *dotted line*.

In the O state, retinal adopts the all-*trans* configuration and Na^+^ is located near the PRSB, stabilized by the carbonyl groups of Asn112, Asp116, Ser70, and the main chain of Val67. They proposed that flipping of the Asn112 side chain is a key motion enabling unidirectional Na^+^ transport. They also proposed that Na^+^ bound at the interfacial ion-binding site near Asp102 contributes to the Na^+^ transport pathway. More recently, time-resolved X-ray free electron laser (TR-XFEL) measurements with femtosecond-to-millisecond temporal resolution revealed sequential structural changes during the KR2 photocycle (22). These results indicated that the transported Na^+^ is transiently coordinated between Asn112 and Asp251 in the O intermediate (Fig. 1*C*) and then coordinated between Asn106 and Glu11 near Arg243—homologous to Glu194 of BR—before release to the cytoplasm (Fig. 1*D*). Their time-resolved FTIR data also tentatively assigned a band at (+) 1688 cm^−1^ to the C=O stretching vibration of Asn112, although they did not verify the assignment using a mutant protein. In addition, the (+) 1689-cm^−1^ band in the light-induced difference FTIR spectrum between the photostationary and dark states of a sodium-pumping rhodopsin from *Gillisia limnaea* (*Gl*NaR) was also proposed to originate from Asn112 (23).

While three-dimensional structures of KR2 are now well established, dynamic structural changes—particularly retinal torsion and hydrogen-bond rearrangements associated with Na^+^ transport—remain insufficiently understood. Notably, two O intermediates with distinct Na^+^-binding states were recently detected in *Ia*NaR by global fitting of transient absorption spectra (24). In a previous time-resolved FTIR spectroscopy of KR2 and *Nonlabens dokdonensis* rhodopsin 2 (NdR2), the obtained data were analyzed by use of a single O intermediate, which contained a distorted 13-*cis* chromophore (25). Presence of a single O intermediate was also the case for another time-resolved FTIR spectroscopy of KR2 (26). To investigate structural dynamics of KR2 in more detail, we applied time-resolved step-scan FTIR spectroscopy to KR2 under different sodium concentrations. Singular value decomposition (SVD) and global fitting successfully resolved four spectrally distinct states, including two O intermediates. Comprehensive analysis using isotopically labeled proteins and mutants enabled assignment of the transient Na^+^-bound state of Asn112 in the O_1_ intermediate. We further identified characteristic protein-backbone and retinal structural changes associated with Na^+^ pumping and proposed that Na^+^ binding to Asp102 accelerates Na ^+^ release during O_2_ decay especially at high Na ^+^ concentration. Based on these analyses together with transient absorption data and kinetic parameters, we discuss a mechanistic model for Na^+^ transport by KR2.

## Results

### Assignment of intermediate states to SAS spectra obtained from time-resolved FTIR spectra of KR2 WT by comparison with transient visible absorption changes

First, we investigated the light-induced transient absorption changes of hydrated KR2 films at 605, 520, and 420 nm, which monitor the formation and decay of red-shifted intermediates, the dark state, and blue-shifted intermediates, respectively (15). The time traces of the hydrated film closely resembled those of the solubilized sample (Fig. S2*a*). Global fitting of the three traces yielded five time constants: τ_1_ = 15 μs, τ_2_ = 160 μs, τ_3_ = 785 μs, τ_4_ = 2.5 ms, and τ_5_ = 7.2 ms. Based on the previous studies of solubilized KR2, τ_2_, τ_3_, τ_4_, and τ_5_ correspond to the decay of the K, L/M, early O (O_1_), and late O (O_2_) intermediates, respectively. These results confirm that the photocycle of the hydrated film is essentially identical to that of the solubilized and lipid-reconstituted samples.

**Figure 2:**
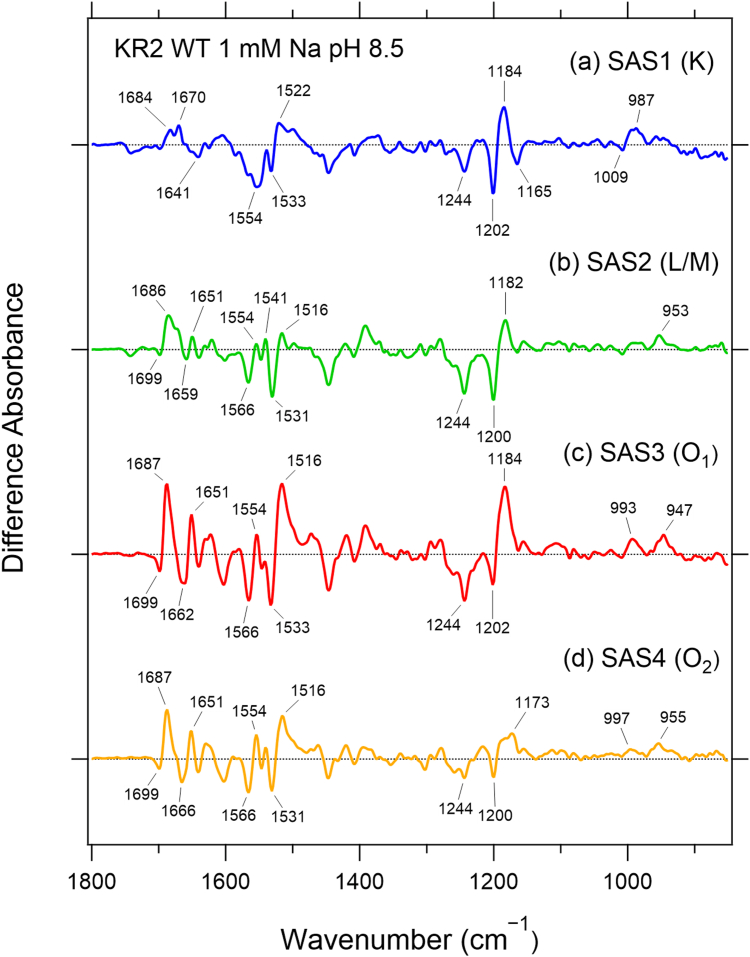
**SAS spectra obtained from time-resolved step-scan FTIR measurements in the 1800–850 cm^−1^ region using global exponential fitting**. Each spectrum corresponds to the intermediate indicated in parentheses after the SAS number: (*a*) K, (*b*) L/M, (*c*) O_1_, and (*d*) O_2_.

Next, we recorded time-resolved step-scan FTIR spectra and performed SVD followed by global exponential fitting. The time-resolved dataset was decomposed into four spectrally distinct components, yielding four species associated spectra (SAS) —SAS1, SAS2, SAS3, and SAS4—as shown in Fig. 2. Their associated time constants, 98 μs, 740 μs, 2.1 ms, and 8.0 ms, agree well with t_2_–t_5_ derived from the transient absorption measurements (Fig. S2*b*). We therefore assigned SAS1, SAS2, SAS3, and SAS4 to the K, L/M, O_1_, and O_2_ intermediates, respectively. The characteristic retinal vibrational signatures described below further support these assignments. The spectral features of SAS1, SAS2, and SAS3 are similar to the previous time-resolved FTIR spectra of K, L/M, and O intermediates, respectively (25).

Before discussing detailed spectral features of each state, we summarize the key characteristics of the SAS1–4 spectra (Fig. 2).

#### SAS1 (K intermediate)

The spectral features of SAS1 closely match those of the K − dark difference spectrum measured at 77 K (27), particularly in the C–C stretching and hydrogen-out-of-plane (HOOP) regions (Fig. S2*c*). The bands at (−) 1202/(+) 1184 cm^−1^ represent the all-*trans* → 13-*cis* isomerization typical of microbial rhodopsins. The (+) 987 cm^−1^ band exhibits a deuterium shift, indicating retinal twist near the PRSB in the K intermediate (27).

The C=C stretching pair (−) 1533/(+) 1522 cm^−1^ corresponds to accumulation of the red-shifted K state, consistent with previous reports (15, 27, 28, 29). In addition, SAS1 contains several room temperature–specific bands—(−) 1554, (+) 1670, (+) 1684, and (−) 1741 cm^−1^—that are absent at 77 K, suggesting larger protein-backbone structural changes at room temperature. Based on low-temperature FTIR studies (27, 30), the (−) 1641 cm^−1^ band shifts to 1624 cm^−1^ in D_2_O and is assigned to the C=N stretch of the PRSB, consistent with the 77 K data.

#### SAS2 (L/M intermediate)

SAS2 exhibits (+) 1182 cm^−1^ in the C–C stretching region and (+) 1541, (+) 1516 cm^−1^ in the C=C region. These features indicate that the 13-*cis* retinal chromophore with the PRSB is present and that the slightly blue-shifted (L) intermediate coexist with the largely blue-shifted (M) one, which was detected by visible transient absorption, in this time window, consistent with SAS2 representing the L/M − dark difference spectrum.

#### SAS3 (O_1_ intermediate)

SAS3 shows (+) 1184 and (+) 1516 cm^−1^, indicating a red-shifted intermediate containing 13-*cis* retinal. The HOOP bands (+) 993, (+) 947 cm^−1^ have larger amplitudes than in SAS2, suggesting stronger retinal torsion in O_1_. Amide-region bands reach their maximum amplitude in SAS3, implying that protein-backbone structural changes peak in the O_1_ state. Previous studies have shown that Na ^+^ binds inside KR2 during the O state (21, 22, 28), and the large backbone rearrangement in SAS3 is likely triggered by Na^+^ binding. The (−) 1699/(+) 1687 cm^−1^ bands show slight D_2_O shifts and increase in amplitude at higher Na ^+^ concentrations as discussed later, suggesting that they originate from transient Na^+^ coordination by Asn or Gln side-chain C=O groups in the O_1_ intermediate. This hypothesis motivated the isotopically labeled and mutant analyses described later.

#### SAS4 (O_2_ intermediate)

SAS4 features a (+) 1173 cm^−1^ band, indicating an all-*trans* retinal configuration (31), consistent with one interpretation of the O-state crystal structure (21). The HOOP bands (+) 997, (+) 955 cm^−1^ are smaller than in SAS3, indicating reduced retinal torsion in the all-*trans* state. Amide-region amplitudes are smaller than in SAS3, suggesting relaxation of the protein structure toward the dark state. These observations are consistent with Na ^+^ no longer being bound near the PRSB in O_2_.

#### Additional observations

Unlike a previous time-resolved FTIR study (26), we did not detect C=O stretching features attributable to the protonation of a carboxylic acid. We infer that Asp116 does not stably accept a proton in the photocycle of lipid-reconstituted KR2. Furthermore, the time constants obtained from the D_2_O-hydrated sample exhibited no significant kinetic isotope effect (Fig. S2*b*), indicating that proton transfer is not rate-limiting in the KR2 photocycle. It should be mentioned that the absence of a detectable protonation signal for Asp116 in this study does not necessarily mean that this residue is not transiently protonated during the photoreaction. In SAS2, the equilibrium between the L and M intermediates would be relatively shifted toward the L state, resulting in only a minor contribution from the M intermediate. In addition, during formation of the O_1_ intermediate in SAS3, Asp116 may already be deprotonated as a consequence of Na^+^ binding near Asp116.

### Precise band assignment of SAS spectra using isotopically labeled proteins and mutants

To clarify the origin of the characteristic FTIR bands in the intermediate states, we analyzed the spectra of KR2 WT together with the N112D mutant, uniformly ^13^C- and ^15^N-labeled KR2, and KR2 containing site-selectively deuterated retinal. Time constants determined from the time-resolved FTIR spectra of all labeled proteins are summarized in Table S1 and were comparable to those of WT.

#### Assignment of the 1687 cm^−1^ band using the N112D mutant

**Figure 3:**
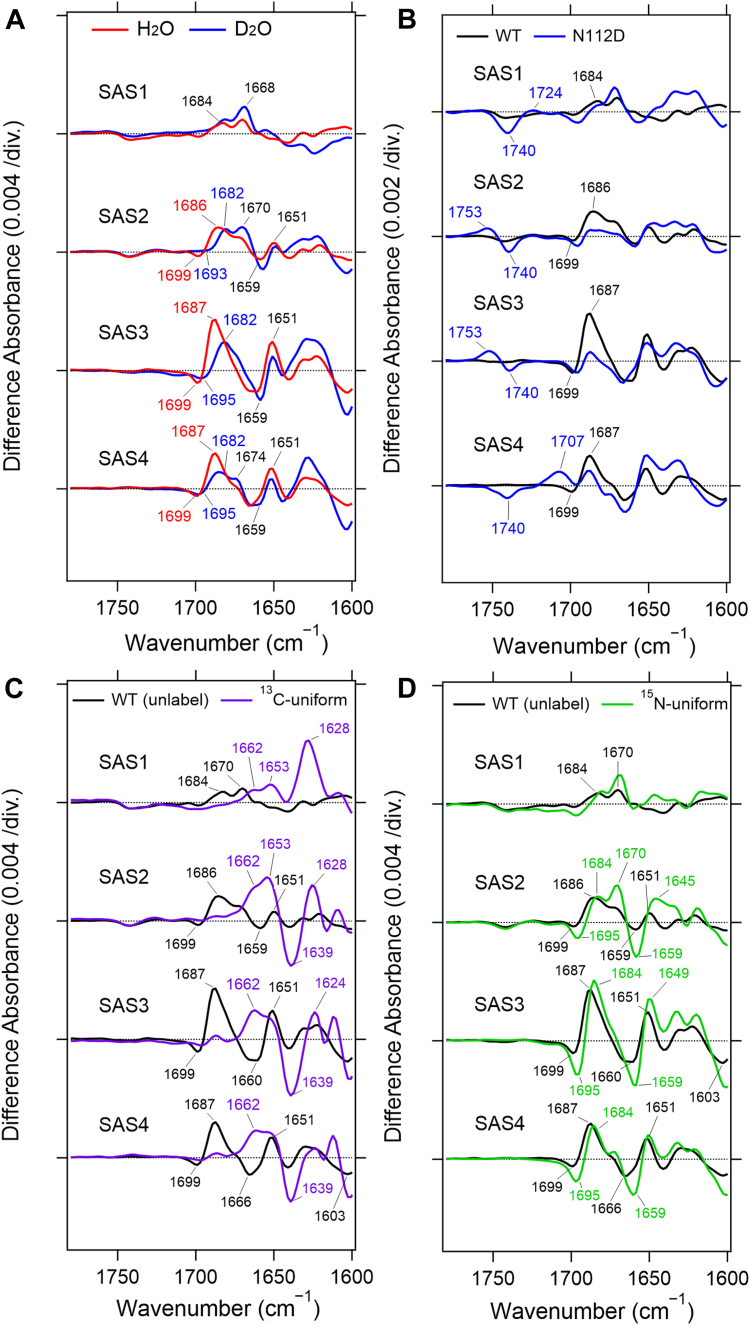
**SAS spectra obtained from time-resolved step-scan FTIR measurements in 1780–1600 cm^−1^**. *Black* and *red* lines (*A*) represent SAS spectra of unlabeled KR2 hydrated with H_2_O and D_2_O, respectively. *Light blue* (*B*), *purple* (*C*), and *green* (*D*) lines represent SAS spectra of N112D mutant, ^13^C-uniformly labeled KR2, and ^15^N-uniformly labeled KR2, respectively. The measurements in *B*–*D* were performed with the samples hydrated with H_2_O.

As shown in Fig. 3*A*, a peak at 1687 cm^−1^ gradually increased its intensity from SAS2 to SAS3, which exhibits the maximum. Then, it decreases the intensity in SAS4. The band at 1687 cm^−1^ is sensitive to H/D exchange, thus, it would not be an amide I band originated from the protein backbone. Interestingly, the band increased in amplitude at higher Na^+^ concentration as shown later.

Given reports that Asn112 coordinates Na^+^ in the O intermediate (21, 22), we hypothesized that the (+) 1687 cm^−1^ band originates from the side-chain C=O stretch of Asn112. To test this, we examined the N112D mutant, which exhibited decreased Na^+^-pumping activity (15). Global fitting resolved four states, and the overall photocycle was approximately 20-fold slower than in WT (Figs. 3*B*, S3) as expected from its low Na^+^-pumping activity. Importantly, the (+) 1687 cm^−1^ band completely disappeared in SAS3 of N112D, and instead new bands appeared at (−) 1740 and (+) 1753 cm^−1^. These results allow us to assign the (+) 1687 cm^−1^ band definitively to the C=O stretching vibration of Asn112, while the newly emerging bands are attributed to protonated Asp112 in the mutant.

The observation that Asp112 is protonated in the N112D mutant, and that its C=O stretching vibration appears at a relatively high frequency, was unexpected. As shown in Fig. 1, *B* and *C*, Asn112 serves as a Na^+^-coordinating residue in WT KR2, implying a strong interaction with the cation. Under such conditions, one might expect the Asp112 side chain in the N112D mutant to be deprotonated and negatively charged due to the proximity of the positive charge of Na^+^.

However, our results strongly suggest that Asp112 remains protonated, whereas Asp116 or Asp251 is deprotonated. This interpretation is reasonable because no other negatively charged residues are located nearby; therefore, if Asp112 does not provide a negative charge through deprotonation, Asp116 or Asp251 must be deprotonated to neutralize the charge of the bound Na^+^.

These findings indicate that, even in WT KR2, Asp116 or Asp251 likely plays the primary role in stabilizing the Na^+^ ion, whereas Asn112 contributes more supportive interactions. The present results therefore suggest distinct functional roles of the two residues in Na^+^ coordination: Asp116 or Asp251 provides the main electrostatic stabilization, while Asn112 assists as a secondary coordinating site.

The (−)1699 cm^−1^ band did not show a significant shift in N112D, indicating that it originates from a residue other than Asn112. The negative band corresponding to Asn112 in the dark state remains unclear; earlier results showed that Asn112 does not participate in the hydrogen-bonding network around the PRSB in the dark state, suggesting vibrational heterogeneity that prevents a distinct negative feature.

In SAS4, the bands at (−) 1740 and (+) 1707 cm^−1^ likely correspond to the C=O stretching vibration of protonated Asp112. Their frequency changes across intermediates suggest that the hydrogen bond of Asp112 in the N112D mutant is weakened in the L/M and O_1_ states, then strengthened again in the O_2_ state, possibly due to tighter Na^+^ interaction that may contribute to the 20-fold slower decay of O_2_ in the N112D mutant.

#### Analysis of amide I bands using ^13^C- and ^15^N-labeled KR2

To further understand protein-backbone structural changes during the photocycle, we analyzed uniformly ^13^C-labeled (Fig. 3*C*) and ^15^N-labeled KR2 (Fig. 3*D*). The (−) 1741 cm^−1^ lipid-associated band showed no isotopic shift, confirming its assignment to lipid C=O stretching and indicating that local protein movements in the L/M intermediate perturb the surrounding lipid environment. In the ^13^C-labeled sample, the band pairs at (−) 1659/(+) 1651 cm^−1^ in SAS2, (−) 1660/(+) 1651 cm^−1^ in SAS3, (−) 1666/(+) 1651 cm^−1^ in SAS4 shifted clearly, allowing assignment to amide I vibrations (Fig. 3*C*). These bands were retained in SAS2–4 of the ^15^N-labeled sample, which support the assignment of the amide I bands (Fig. 3*D*).

The frequency shifts of the amide I bands indicate that the conformation of the α-helix in the intermediates is altered relative to that in the dark state. Consistent with this, SAS3 (O_1_) exhibits the largest amplitudes of these amide bands, supporting the conclusion that the O_1_ intermediate features the largest protein-backbone rearrangement, driven by Na^+^ entry into the ion-binding site.

### Retinal C–C stretching vibrations and their assignments

The peak positions of the retinal C–C stretching vibration bands provide information about the isomeric state of the chromophore. Stable isotope–labeled samples in which specific hydrogens on retinal are replaced with deuterium are particularly useful for assigning individual C–C stretching bands.

**Figure 4:**
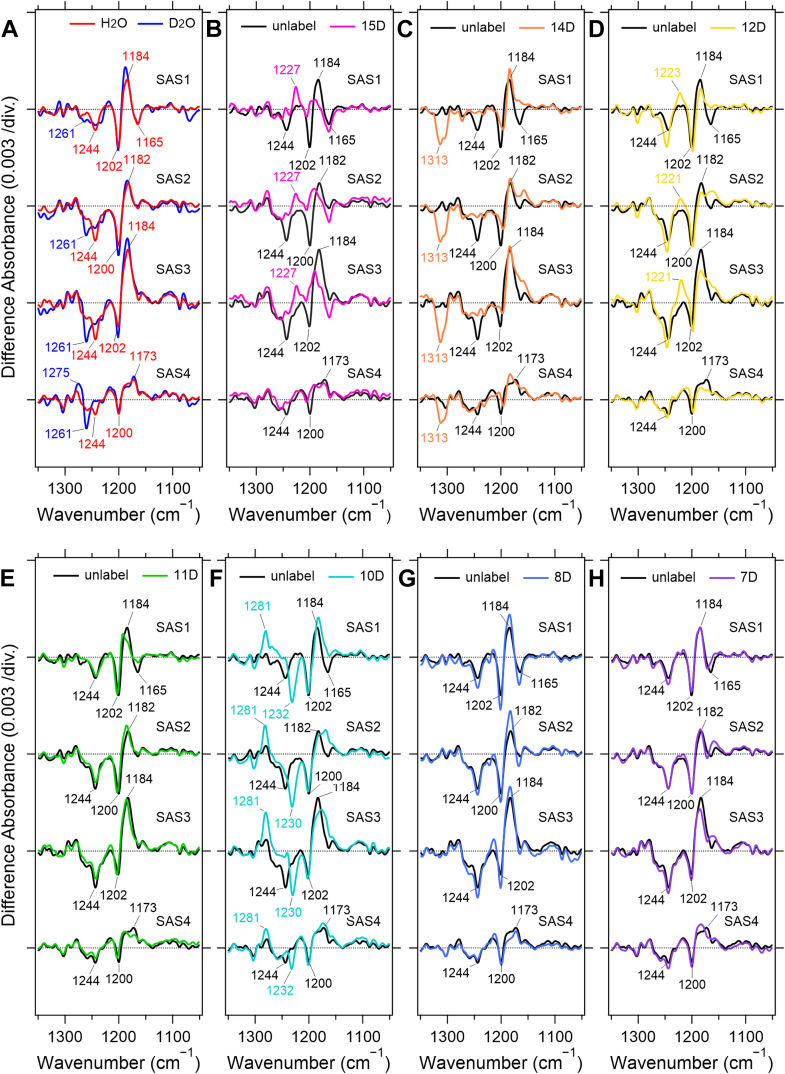
**SAS spectra calculated from time-resolved step-scan FTIR measurements in 1350–1050 cm^−1^**. *Black* and *red* lines in (*A*) represent SAS spectra of unlabeled KR2 hydrated with H_2_O and D_2_O, respectively. SAS1–4 spectra of the isotope-labeled retinal deuterated at each carbon atom are shown in (*B*−*H*) with a different color (15-D, *magenta*; 14-D, *orange*; 12-D, *yellow*; 11-D, *green*; 10-D, *cyan*; 8-D, *light blue*; and 7-D, *purple*). The spectra (*B*−*H*) were recorded with the samples hydrated with H_2_O.

Fig. 4*A* shows SAS1–4 obtained in D_2_O, where the Schiff-base hydrogen is replaced by deuterium. Fig. 4, *B*–*H* show SAS1–4 obtained from time-resolved FTIR measurements of WT KR2 reconstituted with retinals bearing different site-specific deuterium substitutions. In each difference spectrum, the negative bands correspond to the C–C stretching vibrations of retinal in the dark state. Their peak positions are similar to those observed in BR (32), indicating that retinal adopts the all-*trans* configuration in the resting state.

The 1244 cm^−1^ band shifts to 1261 cm^−1^ upon deuteration of the Schiff-base hydrogen. In BR, this band is assigned to the C_12_–C_13_ stretching mode, and its high-frequency shift in the C_14_–D retinal reconstituted sample arises from its coupling with the in-plane bending vibration of C_14_–H. The same behavior is observed in KR2, where the band shifts to 1313 cm^−1^, confirming its assignment to the C_12_–C_13_ stretching vibration. The 1200 cm^−1^ band is assigned to the C_14_–C_15_ stretching mode in BR (32), based on its strong sensitivity to C_15_–H deuteration, and a similar response is observed in KR2. Likewise, the 1165 cm^−1^ band, assigned to the C_10_–C_11_ stretching mode in BR, shows substantial changes upon deuteration of C_10_–H or C_12_–H, which is also consistent with the KR2 data.

On the positive side of the spectra, SAS1–4 show the C–C stretching vibrations of the corresponding intermediates. A strong positive band near 1184 cm^−1^ is commonly observed in SAS1–3, which is characteristic of the 13-*cis* configuration. In contrast, SAS4 exhibits a band at 1173 cm^−1^, shifted to a lower frequency, which is indicative of the all-*trans* configuration.

This band represents a vibrational mode involving conjugated stretching motions across several single bonds—including C_10_–C_11_, C_12_–C_13_, and C_14_–C_15_—as evidenced by the appearance of decoupled peaks at 1227, 1223, and 1281 cm^−1^ in the C_15_–D, C_12_–D, and C_10_–D samples, respectively. Interestingly, such decoupled peaks are not clearly observed in SAS4, reinforcing the conclusion that SAS4 differs from SAS1–3 and that the retinal in SAS4 adopts the all-*trans* configuration.

### Retinal HOOP vibrations and their assignments

Next, we analyzed the HOOP (hydrogen-out-of-plane) bands, which provide information about the degree of twisting of the retinal chromophore in rhodopsins. The intensity of each HOOP band reflects the extent of retinal torsion at the corresponding site, and deuterated retinal analogs are useful for assigning these modes.

**Figure 5:**
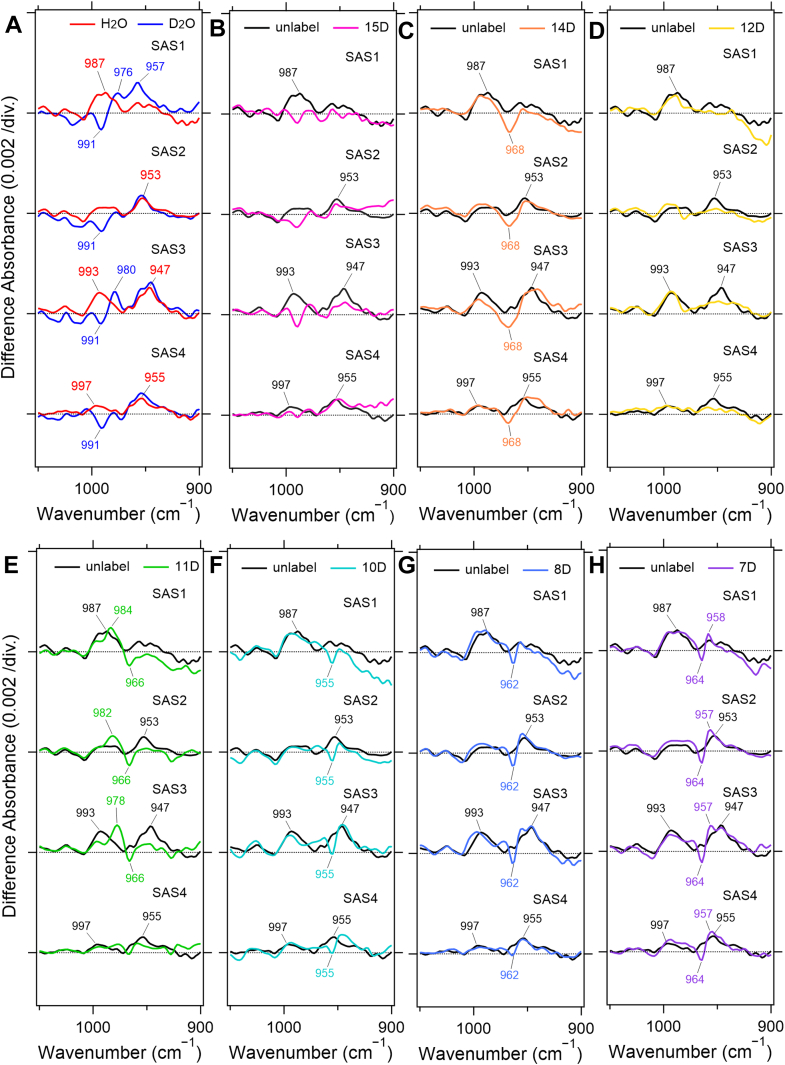
**SAS spectra obtained from time-resolved step-scan FTIR measurements in 1050–850 cm^−1^**. *Black* and *red* lines (*A*) represent SAS spectra of unlabeled KR2 hydrated with H_2_O and D_2_O, respectively. SAS1–4 spectra of the isotope labeled retinal deuterated at each carbon atom are shown in (*B*−*H*) with a different color (15-D, *magenta*; 14-D, *orange*; 12-D, *yellow*; 11-D, *green*; 10-D, *cyan*; 8-D, *light blue*; and 7-D, *purple*). The spectra (*B*−*H*) were recorded with the samples hydrated with H_2_O.

A distinct positive band at 987 cm^−1^ was observed in SAS1, and its intensity decreased in SAS2. This band downshifted upon deuteration of the PRSB (Fig. 5*A*). The 987 cm^−1^ HOOP band in SAS1 and SAS2 disappeared in the spectra of the C_15_–D retinal (Fig. 5*B*), allowing us to assign it to the C_15_–H HOOP vibration.

In SAS3, this band reappeared with increased intensity and slightly shifted to 993 cm^−1^, followed by reduced intensity in SAS4. As in SAS1 and SAS2, these bands in SAS3 and SAS4 were also assigned to the C_15_–H HOOP vibration (Fig. 5*B*). The upshift of the C_15_–H HOOP from 987 to 993 cm^−1^ suggests that the retinal polyene chain adopts different twisting geometries in the early (K and L/M) and later (O_1_ and O_2_) intermediates. The decrease in C_15_–H intensity in SAS2 is partly due to the contribution of the M state, which exhibits weaker retinal-associated vibrational signals. In contrast, the reduced intensity in SAS4 likely reflects the less twisted all-*trans* configuration in the O_2_ intermediate compared with the 13-*cis* configuration in O_1_.

A separate HOOP band at 953 cm^−1^ appeared in SAS2, with no deuterium shift, and shifted to 947 cm^−1^ in SAS3 and 955 cm^−1^ in SAS4. These bands disappeared in the spectra of C_12_–D and C_11_–D retinals (Fig. 5, *D* and *E*) but remained in the other deuterated analogs (Fig. 5, *C*, *F*–*H*). We therefore assigned them to the HOOP vibrations of HC_11_ = C_12_H.

Taken together, the C_15_–H HOOP band showed higher amplitude in SAS1 and SAS3, whereas the HC_11_ = C_12_H HOOP band showed higher amplitude in SAS2 and SAS3. The amplitudes of both HOOP bands in SAS3 increased at higher Na ^+^ concentrations (Fig. S4), suggesting that torsion of the retinal polyene chain is enhanced by Na^+^ binding near the Schiff base.

Based on these observations, the changes in retinal torsion during the photocycle can be summarized as follows: (1) In the K intermediate, torsion near the PRSB increases upon isomerization to the 13-*cis* configuration. (2) In the L/M intermediates, torsion near C_11_ and C_12_ and near the Schiff base becomes slightly relaxed. (3) In the O_1_ intermediate, Na ^+^ binding near the PRSB induces twisting that extends from C_11_ to C_15_. (4) In the O_2_ intermediate, the twisted structure relaxes as retinal returns to the all-*trans* configuration.

### Na^+^ concentration dependence of decay time constants of the intermediates

**Figure 6:**
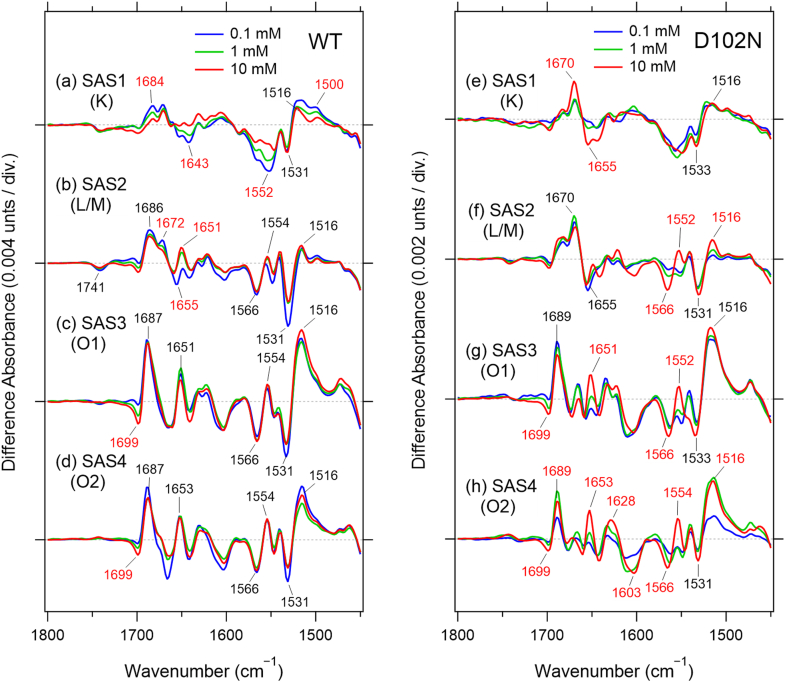
**Na ^+^ -concentration dependency of SAS spectra of WT (*left*) and D102N (*right*) obtained from time-resolved step-scan FTIR measurements in 1800–1450 cm^−1^**. The SAS spectra corresponding to each intermediate are indicated in parentheses after the SAS number: (*a*, *e*) K, (*b*, *f*) L/M, (*c*, *g*) O_1_, and (*d*, *h*) O_2_. The spectra were obtained with the hydrated films prepared from the samples in different Na ^+^ concentrations (*blue*, 0.1 mM; *green*, 1 mM; *red*, 10 mM). The effective Na^+^ concentrations would be ∼100 times higher due to condensation during the film formation. The samples were hydrated with H_2_O.

**Figure 7:**
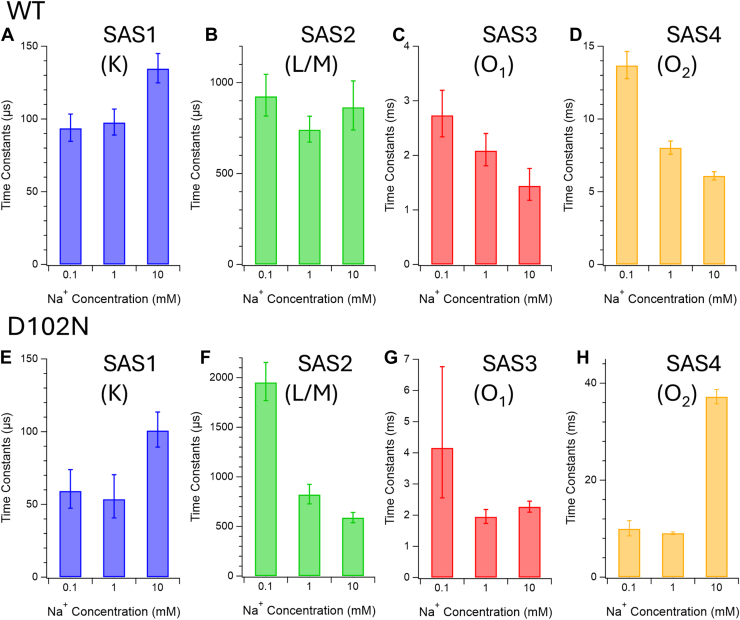
**Time constants of WT (*upper* graphs) and D102N (*lower* graphs) calculated from time-resolved step-scan FTIR measurements on the hydrated films prepared from the samples in 0.01 to 10 mM Na^+^**. The effective Na^+^ concentrations would be ∼100 times higher due to condensation during the film formation. The bar graphs corresponding to each intermediate are indicated in parentheses after the SAS number: (*A*, *E*) K, (*B*, *F*) L/M, (*C*, *G*) O_1_, and (*D*, *H*) O_2_. The error bars are obtained from global exponential fitting on each data set.

To analyze structural changes arising from interactions between Na^+^ and the protein, we measured and compared time-resolved FTIR spectra (Fig. 6(*left*), Fig. S4) and visible transient absorption changes (Fig. S5) as a function of Na^+^ concentration. Hydrated films were prepared from proteoliposome suspensions in buffers containing 0.1 to 10 mM NaCl, and the corresponding time constants are summarized in Table S2. Because of sample condensation during film preparation, the effective Na^+^ concentration in the hydrated films is estimated to be approximately 100-fold higher than that in the original suspension (33). Time constants obtained from time-resolved FTIR and transient absorption measurements are compared in bar graphs (Fig. 7, *A*–*D*, Fig. S5*b*).

To assess the effect of Na^+^ binding at the extracellular Na^+^-binding site, including Asp102, on the photocycle, we also investigated the Na^+^ concentration dependence of decay time constants in the D102N mutant (Fig. 6 (*right*) and Fig. S6). This mutation drastically reduces the Na^+^-binding affinity at the extracellular site while largely preserving Na^+^-pumping activity (16).

#### SAS1 (K intermediate)

SAS1 (K) spectra of WT showed very similar spectral features across all Na^+^ concentrations, except in the amide I and II regions (Figs. 6*a*, S4). The bands at (+) 1684/(−) 1641 cm^−1^ and (−) 1552/(+) 1500 cm^−1^, which are characteristic of time-resolved FTIR spectra compared with low-temperature FTIR spectra of the K intermediate (Fig. S2*c*), exhibited clear Na^+^ concentration dependence. These observations indicate that structural changes in the K intermediate are modulated by Na^+^ binding at the initial extracellular site near Asp102.

Notably, such Na^+^-dependent changes in the amide I and II bands were not observed in SAS1 of the D102N mutant (Fig. 6*e*), supporting the conclusion that an auxiliary Na^+^ at the extracellular binding site induces protein structural modifications already in the K intermediate. In contrast, additional amide I bands at (+) 1670 and (−) 1655 cm^−1^ appeared prominently in the D102N mutant prepared from the 10 mM Na ^+^ condition, which may reflect early release of weakly bound Na^+^ from the extracellular site.

#### SAS2 (L/M intermediate)

SAS2 (L/M) spectra exhibited slight Na^+^ concentration–dependent differences, particularly under 0.1 mM Na^+^ condition. An increase in the (−) 1531 cm^−1^ band and a decrease in the (+) 1182 cm^−1^ band indicate enhanced accumulation of the M intermediate at low Na ^+^ concentration (Figs. 6*b*, S4). This interpretation is supported by visible transient absorption changes at 420 nm (Fig. S5*a*), which show a positive feature around 10^−4^ s.

The increase of the (+) 1672 and (−) 1655 cm^−1^ bands, which accompanies enhanced M-state formation, may suggest Na^+^ release from the extracellular binding site, similar to that observed in the K intermediate of the D102N mutant under 10 mM Na^+^ condition. These bands were also detected in SAS2 of the D102N mutant and may be associated with protein backbone rearrangements near the extracellular binding site around position 102 in the absence of Na^+^.

#### SAS3 (O_1_ intermediate)

SAS3 (O_1_) spectra of WT did not show pronounced Na^+^ concentration–dependent spectral changes, even though the decay time constants of the O_1_ intermediate strongly depended on Na ^+^ concentration. This observation is unexpected, given that a substrate Na^+^ is incorporated upon formation of O_1_ and remains bound during the O_1_-to-O_2_ transition. These results suggest that another Na^+^ is incorporated at an internal binding site distinct from the substrate Na ^+^-binding site, which may be the extracellular Na^+^-binding site at Asp102.

In contrast, the Na^+^ concentration dependence in the D102N mutant was markedly different. Na^+^ ion at the extracellular auxiliary binding site would therefore be prerequisite for conformational change accompanying incorporation of a substrate Na^+^ at this stage. In the D102N mutant, the amide I and II bands at (+) 1651 and (+) 1552 cm^−1^, induced by O_1_ formation, exhibited strong Na^+^ concentration dependence. The SAS3 spectrum of the D102N mutant only under 10 mM Na^+^ condition shows similar spectral change to those of WT, which implies that the D102N mutant prepared under 10 mM Na^+^ condition binds an auxiliary Na^+^ at the extracellular binding site at the dark state as similarly to WT. In addition, the (−) 1699 cm^−1^ band showed clear Na ^+^ dependence in both WT and the D102N mutant. As discussed above, this band does not originate from the C=O stretching vibration of Asn112, but may instead arise from an Asn or Gln residue or a carbonyl group of the peptide backbone located near the extracellular Na^+^-binding site close to Asp102, reflecting binding of an auxiliary Na^+^ at the dark state.

#### SAS4 (O_2_ intermediate)

SAS4 (O_2_) spectra were highly similar across Na^+^ concentrations of 0.1, 1, and 10 mM. Nevertheless, the decay time constants of the O_2_ intermediate exhibited clear Na ^+^ concentration dependence (Fig. 7*D*). Because the substrate Na^+^ is released to the extracellular side during decay of the O_2_ intermediate, this release step is not in equilibrium with the external Na^+^ concentration, consistent with the observation that the O_2_ decay becomes faster at higher Na^+^ concentrations.

Binding of an auxiliary Na^+^ at the extracellular site near Asp102 may facilitate substrate Na^+^ release at this stage, as inferred from the dramatic slowing of O_2_ decay observed in the D102N mutant under high Na ^+^ conditions. In the absence of the auxiliary Na ^+^ at the O_2_ intermediate, substrate Na^+^ release becomes equilibrated with the external Na^+^ concentration, and high Na^+^ levels inhibit release from the ion-transport pathway. This interpretation is further supported by the decrease in SAS4 amplitude observed in the D102N mutant under lower Na^+^ conditions. The affinity of Na^+^ at the extracellular site near Asp102 may be weaker in the O_2_ intermediate than that in the dark state. Hence the decay of O_2_ intermediate of the D102N mutant is dramatically slowed down due to absence of Na ^+^ at the extracellular site.

It should be noted that the D102N mutant exhibits Na^+^-pumping activity comparable to that of WT (15). Thus, the auxiliary Na^+^ is not essential for substrate release under normal Na^+^ concentrations. The reduction of the decay of the O_2_ intermediate of the D102N mutant prepared under 10 mM Na ^+^ condition suggests that the auxiliary Na ^+^ at the extracellular site becomes functionally important under high-salinity conditions.

Finally, time-resolved FTIR measurements performed at very low Na^+^ concentration (0.01 mM) revealed spectral features markedly different from those observed at 1 mM Na^+^ (Fig. S7). These spectra more closely resemble those obtained in 1 mM Cs^+^ (Fig. S8), suggesting a shift toward proton-pumping–related structural changes. Because this behavior is beyond the scope of the present study, detailed discussion of these spectral features will be addressed in future work.

## Discussion

### Sequential structural changes during sodium pumping by KR2

**Figure 8:**
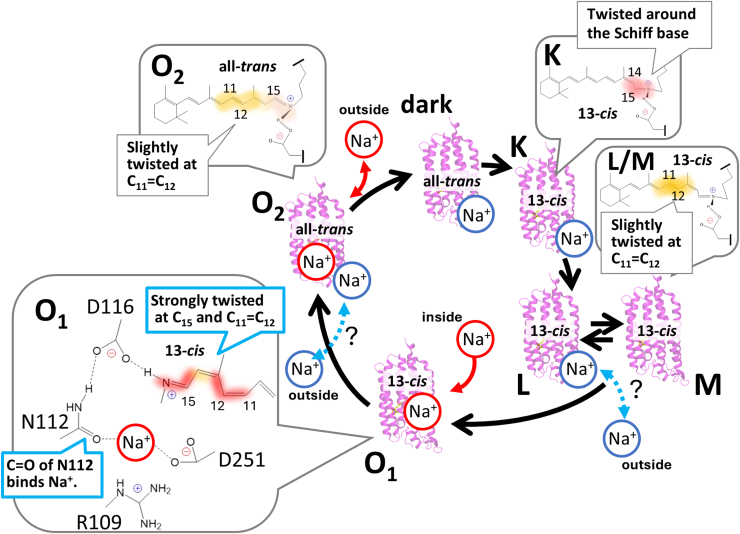
**Schematic model of the sequential structural changes during the photocycle of the sodium-pumping rhodopsin KR2**. A Na^+^ ion bound near Asp102 is shown as a *blue* circle, whereas the substrate Na^+^ ion is shown as a *red* circle. The substrate Na^+^ is incorporated from the cytoplasmic side upon formation of the O_1_ intermediate and is then released to the extracellular side upon return to the dark state. In contrast, the auxiliary Na^+^ is initially bound within KR2, which is assumed to be transiently released to the extracellular side, and subsequently return to the same site during the O_1_-to-O_2_ transition.

Based on our spectroscopic analyses together with recent crystallographic studies, we propose a model for the sequential structural changes during sodium pumping by KR2, involving the K, L/M, O_1_, and O_2_ intermediates, as illustrated in Fig. 8.

Upon formation of the K intermediate, the retinal chromophore isomerizes from the all-*trans* to the 13-*cis* configuration, accompanied by an increased twist around C_15_–H. This conclusion is supported by resonance Raman spectroscopy results (34). Distortion of the retinal chromophore induces structural changes near the PRSB, as reported previously. Specifically, hydrogen bonds involving water molecules near the PRSB are weakened: one water molecule located between Gln123 and the carbonyl group of Lys255, which forms the PRSB (35), and another water molecule near Asp251, the second counterion of the PRSB (30).

Next, upon formation of the L/M intermediate, proton transfer from the Schiff base to the counterion Asp116 has been reported previously (26), although such protonation was not directly confirmed in the present study. The retinal twist around the C_11_ = C_12_ bond and C_15_–H becomes slightly relaxed compared with that in the K intermediate, as indicated by decreased HOOP band intensities. In addition, the (+)1670 and (−)1655 cm^−1^ bands increase in intensity at lower Na ^+^ concentrations, which may suggest that an auxiliary Na^+^ bound on the extracellular side is released during formation of the L/M intermediate. This conformational change is more pronounced in the D102N mutant, which may correlate with its reduced affinity for the auxiliary Na^+^ at the extracellular site.

The O_1_ intermediate is then formed through reprotonation of the Schiff base and uptake of a substrate Na^+^ from the cytoplasmic side. Our spectroscopic data supports that the incorporated Na ^+^ interacts with the C=O group of the Asn112 side chain, in agreement with crystallographic observations (21, 22). In this state, the retinal polyene chain between C_11_ and C_15_ becomes strongly twisted, as evidenced by intense HOOP vibrations. Large-scale protein backbone rearrangements are also suggested by increased intensities of the amide I band at (+)1651 cm^−1^ and the amide II band at (+)1554 cm^−1^.

Because time-resolved measurements were employed in this study, the Na^+^ ion was placed between Asn112 and Asp251 in the schematic model (Fig. 8), consistent with the TR-XFEL structure obtained at 1 ms (Fig. 1*C*). However, the precise Na^+^ position cannot be unambiguously determined from the present data, and it may alternatively reside between Asn112 and Asp116, as suggested by cryogenic trapping structures (Fig. 1*B*).

Finally, upon formation of the O_2_ intermediate, the retinal chromophore thermally isomerizes from 13-*cis* back to the all-*trans* configuration, accompanied by relaxation of the polyene-chain twist between C_11_ and C_15_. Our time-resolved FTIR data demonstrate that decay of the O intermediates is accelerated at higher Na^+^ concentrations, supporting the hypothesis that rebinding of the auxiliary Na^+^ on the extracellular side facilitates release of the substrate Na^+^ from KR2 through electrostatic repulsion. This interpretation is strongly supported by the dramatic slowing of O_2_ decay kinetics observed in the D102N mutant under high-Na^+^ conditions. Interestingly, in *Gl*NaR, a model has been proposed in which, in addition to the substrate Na^+^ ion, an H^+^ is released during formation of the O intermediate and subsequently taken up upon return to the initial state (23). It may be possible that this H^+^ corresponds to Na^+^ in KR2. However, verification of the proposed dissociation and rebinding of auxiliary Na^+^ near Asp102 during the photocycle will require more comprehensive analyses, including systematic mutagenesis, further time-resolved spectroscopic studies, and computational investigations such as molecular dynamics simulations. Furthermore, it remains to be determined whether the Na ^+^ transport mechanism proposed here for KR2 is also applicable to *Ia*NaR, in which two O intermediates were reported as well (24).

## Conclusions

Our spectroscopic analyses revealed four spectrally distinct states in the photocycle of the sodium-pumping rhodopsin KR2, namely K, L/M, O_1_, and O_2_. Among them, the O_1_ intermediate exhibits the largest structural changes, which are induced by Na^+^ binding near the C=O group of the Asn112 side chain. This interaction is accompanied by strong twists of the retinal polyene chain in the region from C_11_ to C_15_.

Our comprehensive analysis suggests that this retinal twisting is specific to the sodium-pumping reaction of KR2 and is not observed in the proton-pumping form. During the transition from O_1_ to O_2_, the retinal chromophore undergoes thermal isomerization from 13-*cis* to all-*trans*, accompanied by Na^+^ binding at the substrate binding site near Asn112. We also propose the hypothesis that an additional Na^+^ binding site on the extracellular side near Asp102 may contribute to the Na^+^-pumping mechanism of KR2. Namely, this auxiliary Na^+^ plays a supportive role in facilitating the release of the substrate Na^+^ ion, particularly under high Na^+^ concentration conditions.

## Experimental procedures

### Preparation of KR2 samples

The expression plasmids were constructed using a synthetic KR2 gene as described previously (36, 37). Mutant plasmids were generated using the QuikChange site-directed mutagenesis kit (Stratagene) according to the standard protocol. KR2 proteins bearing a C-terminal hexahistidine tag were expressed in *Escherichia coli* C41(DE3). Precultures (5–20 ml) were grown in shaker flasks at 37 °C and 180 rpm and then inoculated into 1 L cultures with aeration. When the OD_660_ reached 0.5, protein expression was induced by adding 1 mM IPTG and 10 μM all-*trans* retinal, followed by incubation for an additional 4 h.

Cells (1 L) expressing KR2 WT or mutants were disrupted using a French press, and the membrane proteins were solubilized with 2% n-dodecyl-β-D-maltoside. KR2 was purified by cobalt-affinity chromatography (TALON, TAKARA Bio) as described previously (27). Solubilization and elution were performed at pH 6.5 and 7.0, respectively.

For preparation of KR2 with isotopically labeled retinal, 2 L cultures were grown without adding all-*trans* retinal. Half of the harvested cells were disrupted using a French press, and 10 μmol all-*trans* retinal was added to the stirred membrane suspension, resulting in rapid pigment formation. Solubilization and purification were performed as for the WT protein, yielding 19.8 mg of KR2. From this yield, the amount of added retinal corresponded to approximately 16.7-fold molar excess over KR2 opsin. We assumed that the remaining half of the culture contained a similar amount of opsin.

To minimize the consumption of isotopically labeled retinal, only 2/15 of the remaining half-culture—sufficient to obtain adequate protein for spectroscopy—was used for purification. Prior to solubilization, isotopically labeled retinal was added at a 16.7-fold molar excess over KR2 opsin. Attempts to reduce the degree of excess retinal resulted in significantly poorer purification efficiency.

For preparation of isotopically labeled KR2 proteins, *E*. *coli* C41(DE3) cells were grown in minimal medium (M9). The medium contained either ^13^C-glucose for production of uniformly ^13^C-labeled protein or ^15^N-ammonium chloride for production of uniformly ^15^N-labeled protein.

### Transient visible absorption measurements

Purified KR2 proteins were reconstituted into liposomes composed of POPE and POPG (molar ratio 3:1) at a protein-to-lipid molar ratio of 1:20 by removing 2% n-dodecyl-β-D-maltoside with Bio-Beads (SM-2, Bio-Rad). The reconstituted samples were washed three times with 1 mM NaCl and 2 mM Tris–HCl (pH 8.5). The pellet was then resuspended in the same buffer, and the protein concentration was adjusted to ∼1.3 mg/ml. A 40 μl aliquot was applied onto a BaF_2_ window and gently dried at room temperature.

Measurements were performed using hydrated films prepared by adding ∼6 μl of H_2_O containing 20 to 30% (v/v) glycerol. The hydrated film was covered with another BaF_2_ window separated by a silicone rubber spacer and sealed with Parafilm. The sample was mounted in a temperature-controlled transmission cell holder (TFC-M25-3, Harrick). The temperature during measurements was maintained at 20 °C using a circulating water bath (Alpha RA8, Lauda).

For excitation, each sample was illuminated with the second harmonic (λ = 532 nm) of a nanosecond-pulsed Nd^3+^:YAG laser (INDI40, Spectra-Physics). The laser pulse energy was ∼0.7 mJ per pulse, and the repetition rate was set to ∼2 Hz, sufficiently slower than the photocycle kinetics of KR2 to avoid unintended re-excitation of transient intermediates.

Changes in absorption following photoexcitation were probed using monochromated light from an Xe arc lamp (L9289-01, Hamamatsu Photonics) through two monochromators (S-10, Soma Optics Ltd, Japan). The transmitted probe light was detected with a photomultiplier tube (R10699, Hamamatsu Photonics), and the signal was averaged and recorded using a digital storage oscilloscope (DPO7104, Tektronix).

### Time-resolved step-scan FTIR measurements

Sample preparation was identical to that used for transient absorption measurements. Time-resolved FTIR spectra were recorded on a Vertex 80 spectrometer (Bruker Optics) equipped with a linearized MCT detector as similarly in a previous paper (38). The spectrometer was continuously purged with dry N_2_ gas to minimize sharp absorption peaks originating from atmospheric H_2_O vapor.

Photoreaction was initiated with a 532 nm laser pulse using an Nd:YAG laser (LS-2134UTF, Lotis TII). The excitation energy was adjusted to 1.4 mJ per pulse using neutral-density filters, and the repetition rate was controlled at 2 Hz with an electromagnetic shutter (SH-05, Thorlabs). Synchronization of the FTIR spectrometer with the laser was controlled by a digital delay generator (DG645, Stanford Research Systems). The resulting time and spectral resolutions were 12.5 μs and 4 cm^−^^1^, respectively.

An interferogram matrix I(i,x) was constructed from 999 sampling points (x) corresponding to mirror positions. At each fixed mirror position, light-induced transient signals were co-added five times over up to 10,000 time slices (i) ranging from −228 μs to 124 ms, using the time-resolved step-scan mode. Each interferogram was Fourier-transformed into a single-beam spectrum over the range 1950–850 cm^−1^. The 16 time slices acquired prior to laser excitation were averaged and used as the reference spectrum.

To improve spectral quality and reduce the number of sampling points, infrared light above 2000 cm^−1^ was removed using a long-pass IR filter (LP-5000, IR Systems). For each sample condition, spectra from 12 to 28 independent measurements were averaged.

### SVD and global fitting analysis for time-resolved FTIR spectra

To characterize the observed temporal changes in the time-resolved FTIR spectra, global exponential fitting was performed in combination with SVD using all averaged spectral series and custom MATLAB scripts developed by Dr Víctor Lórenz-Fonfría (39). The number of exponential components was first estimated by globally fitting the most significant abstract time traces (U columns). Abstract spectra (V columns) with large associated singular values (diagonal elements of S) were interpreted as representing genuine spectral changes with amplitudes clearly above the noise level.

Subsequently, the raw experimental data or the SVD-reconstructed data were globally fitted using the same number of exponential components. This procedure yielded decay-associated spectra, also referred to as amplitude spectra, as well as the corresponding SAS, which were generated on the basis of an irreversible and unidirectional sequential reaction model. The experimental data were fitted according to the following expression:

$$\text{Data}\;\left(t\right)\approx \left(\sum_{i}{\text{DAS}}_{i}\;\exp\left(-k_{i}t\right)+offset\right)$$

where *i* denotes the number of resolved intermediates with their respective time constants $k_{i}$. The offset term accounts for an unresolved intermediate whose decay is slower than the temporal window accessible in the experiment.

## Data availability

All representative data are contained within the article and in the supporting information.

## Supporting information

This article contains supporting information.

## Declaration of generative AI and AI-assisted technologies in the writing process

During the preparation of this work, the authors used ChatGPT (OpenAI) in order to improve the English language of the manuscript. After using this tool, the authors reviewed and edited the content as needed and take full responsibility for the content of the publication.

## Conflict of interest

The authors declare that they have no conflicts of interest with the contents of this article.
