# Supplementary material for: Mechanistic insights into Na+ pumping by KR2: Distinct roles of Asp102 and Asn112 coupled with retinal distortion in two O intermediates
Source: J Biol Chem. 2026 Feb 26;302(4):111313. doi: 10.1016/j.jbc.2026.111313 (PMC13129529; doi:10.1016/j.jbc.2026.111313)
Supplement: Supplementary Material [file mmc1.docx]

**Supporting Information**

**Mechanistic insights into Na⁺ pumping by KR2: distinct roles of Asp102 and Asn112 coupled with retinal distortion in two O intermediates**

*Sahoko Tomida^1^, Rei Abe-Yoshizumi^1^, Akimori Wada^2^, Hideki Kandori^1,3^, and Yuji Furutani^1,3^**

^1^Department of Life Science and Applied Chemistry, Nagoya Institute of Technology, Showa-ku, Nagoya 466-8555, Japan

^2^ Laboratory of Organic Chemistry for Life Science, Kobe Pharmaceutical University, Higashinada-ku, Kobe 658-8558, Japan,

^3^OptoBioTechnology Research Center, Nagoya Institute of Technology, Showa-ku, Nagoya 466-8555, Japan

* Address Correspondence to Phone & Fax: +81 52 735 5127, E-mail: furutani.yuji@nitech.ac.jp

List of the contents in the supporting information

**Figure S1**: X-ray crystal structures of KR2 in the dark state

**Figure S2**: Comparison of time-resolved FTIR, visible transient absorption spectroscopy, and low-temperature FTIR measurements

**Figure S3**: SAS1–4 spectra obtained from time-resolved FTIR spectroscopy of the N112D mutant

**Figure S4**: SAS1–4 spectra obtained from time-resolved FTIR spectroscopy of WT films prepared from proteoliposome suspensions in buffers containing 0.1, 1, or 10 mM NaCl

**Figure S5**: Na⁺-concentration dependence of visible transient absorption spectroscopy in WT KR2

**Figure S6**: SAS1–4 spectra obtained from time-resolved FTIR spectroscopy of the D102N mutant films prepared from proteoliposome suspensions in buffers containing 0.1, 1, or 10 mM NaCl

**Figure S7**: SAS1–4 spectra obtained from time-resolved FTIR spectroscopy of WT films prepared from proteoliposome suspensions in buffers containing 0.01 and 1 mM NaCl

**Figure S8**: SAS1–4 spectra obtained from time-resolved FTIR spectroscopy of WT films prepared from proteoliposome suspensions in buffers containing 0.01 mM NaCl or 1 mM CsCl

**Table S1**: Time constants obtained from time-resolved FTIR measurements of WT KR2 and N112D mutant

**Table S2**: Time constants obtained from time-resolved FTIR spectroscopy of WT KR2 and the D102N mutant at different Na⁺ concentrations (0.1, 1, and 10 mM)


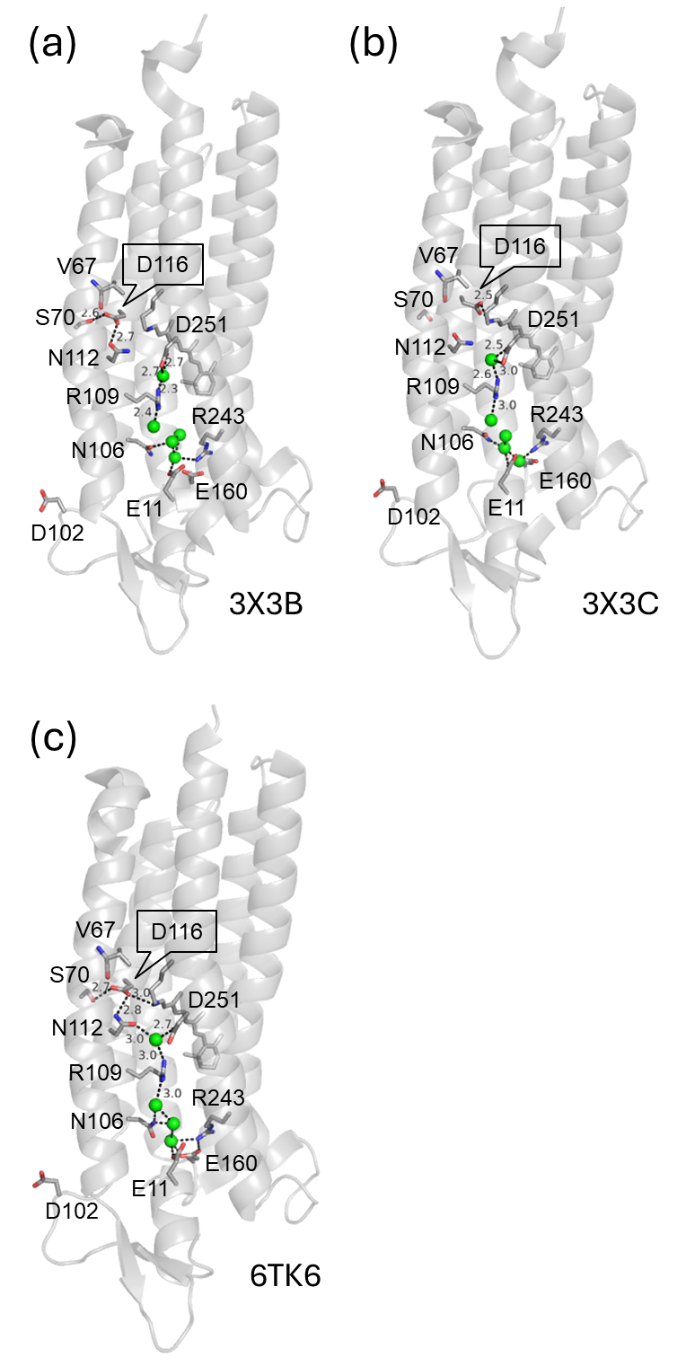


**Figure S1.** X-ray crystal structures of KR2 in the dark state. Crystal structures determined under acidic (a) and neutral pH (b) conditions are shown in the monomeric form within the crystallographic unit. The side chain of Asp116 points toward Asn112 in (a) and toward the protonated retinal Schiff base (PRSB) in (b). The dark-state structure in the pentameric form used for TR-SFX is shown in (c).


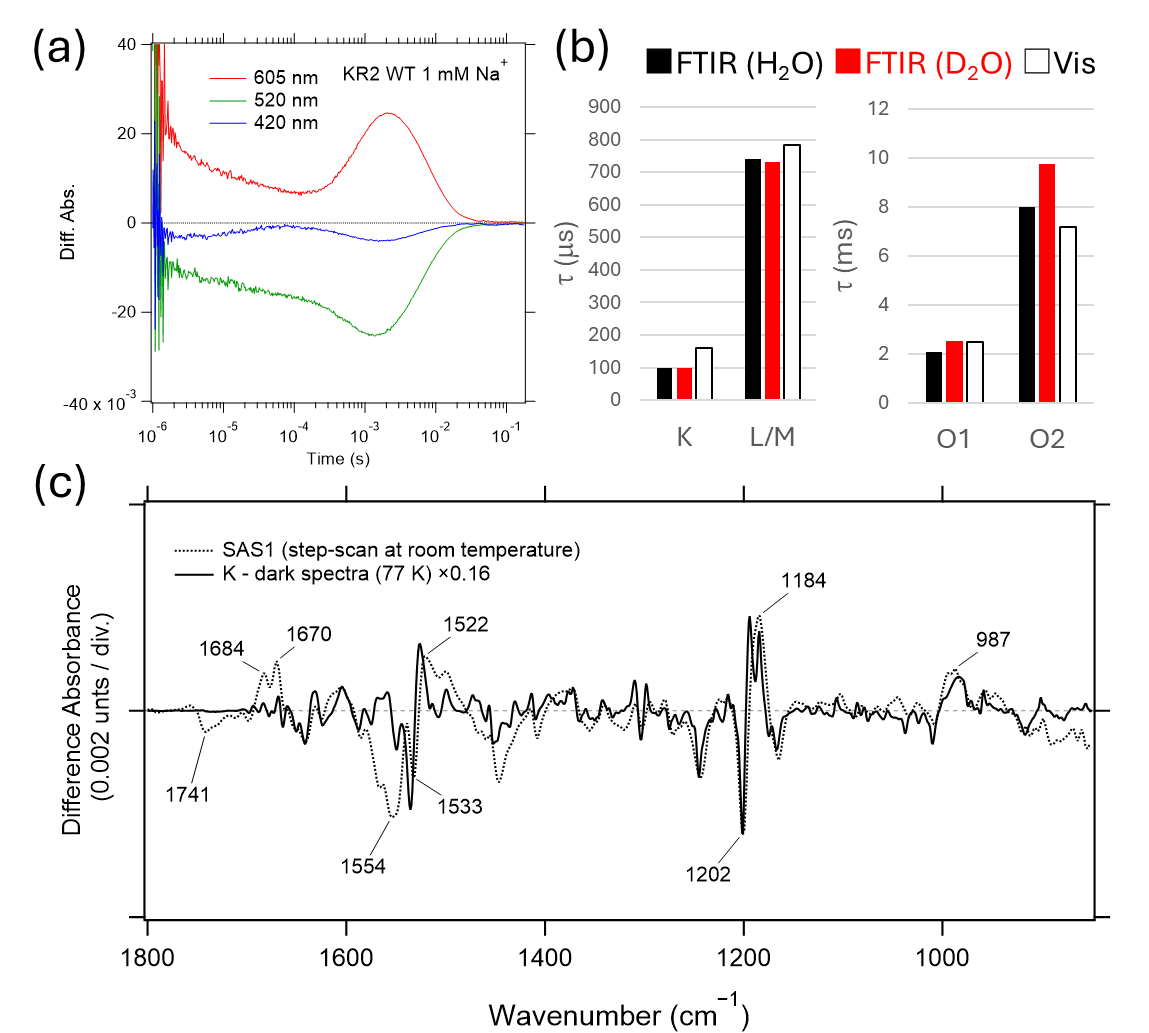


**Figure S2.** Comparison of time-resolved FTIR, visible transient absorption spectroscopy, and low-temperature FTIR measurements. (a) Visible transient absorption traces at 605, 520, and 420 nm recorded for a hydrated film of KR2 WT. (b) Time constants obtained from time-resolved FTIR and visible flash photolysis measurements, shown as bar graphs. (c) Light-induced difference infrared absorption spectra of KR2 WT recorded at 77 K, shown for comparison with the SAS1 spectrum.


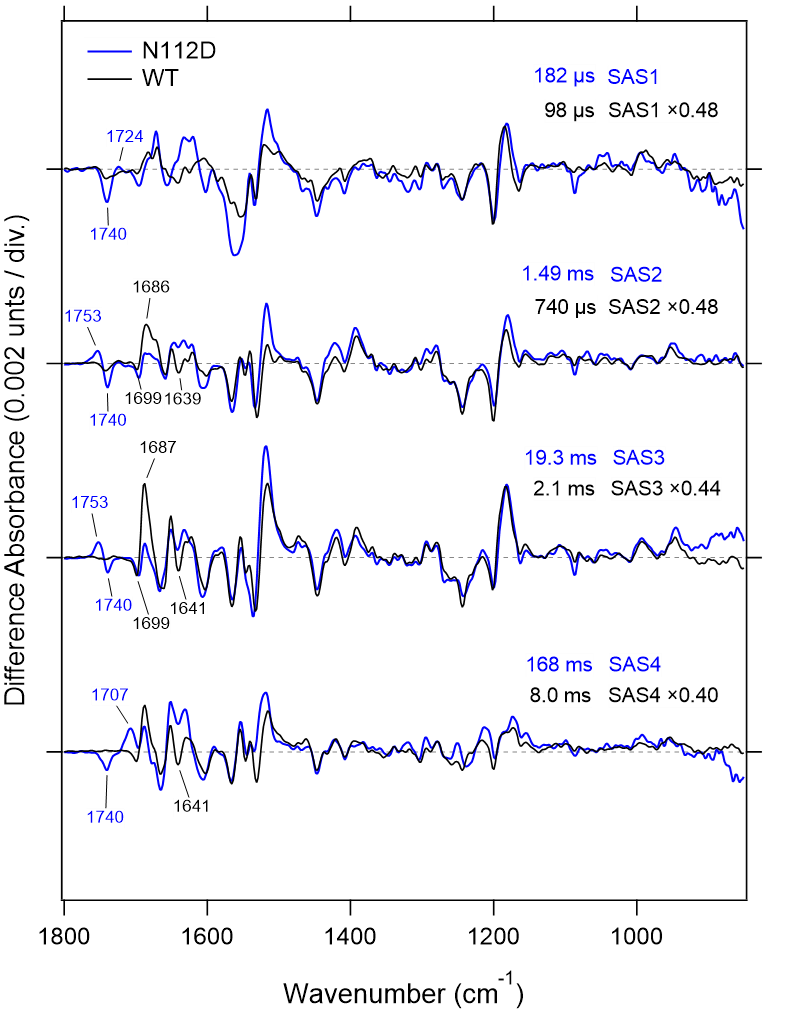


**Figure S3.** SAS1–4 spectra obtained from time-resolved FTIR spectroscopy of the N112D mutant. The spectra are shown in the 1800–900 cm^−1^ region. The associated time constants are indicated for comparison with those of WT. Spectra of the N112D mutant and WT are shown in blue and black, respectively.


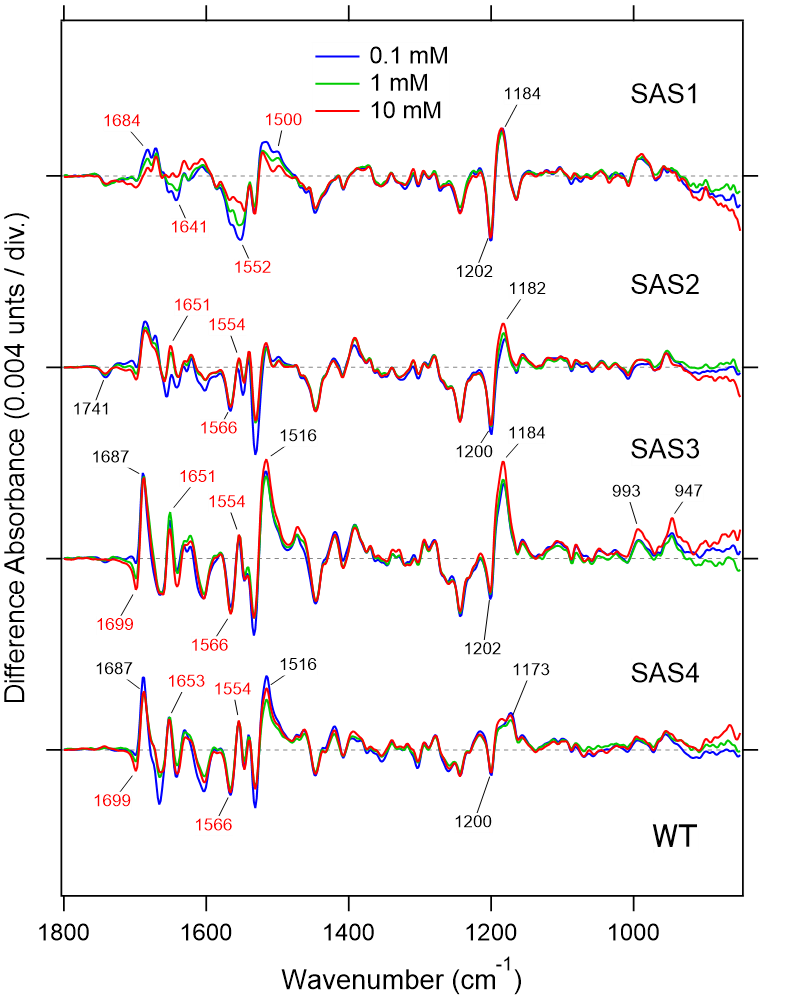


**Figure S4.** SAS1–4 spectra obtained from time-resolved FTIR spectroscopy of WT films prepared from proteoliposome suspensions in buffers containing 0.1, 1, or 10 mM NaCl. Spectra recorded under 0.1, 1, and 10 mM Na⁺ conditions are shown in blue, green, and red, respectively.


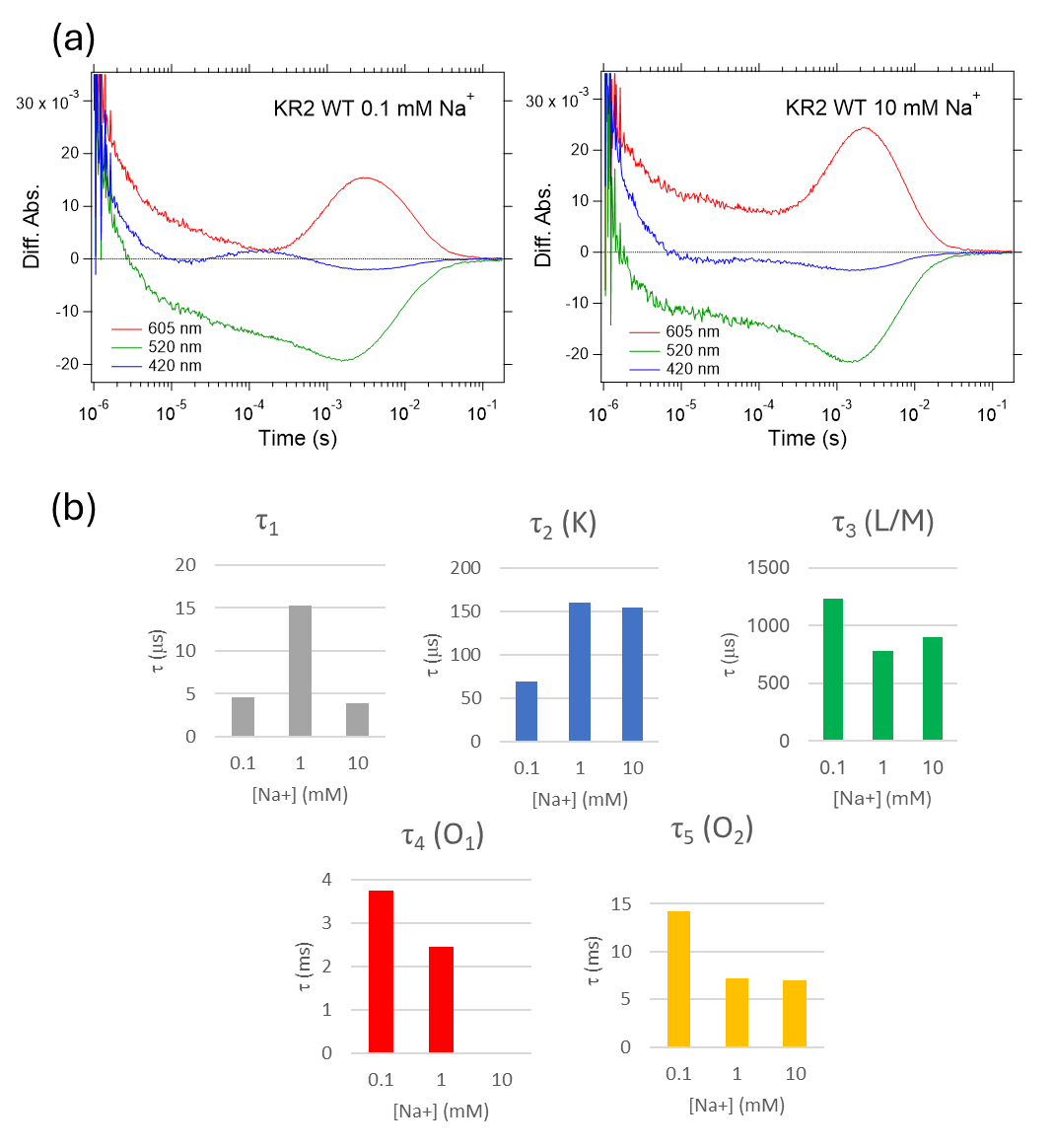


**Figure S5.** Na⁺-concentration dependence of visible transient absorption spectroscopy in WT KR2. (a) Visible transient absorption measurements of WT films prepared from proteoliposome suspensions in buffers containing 0.1 or 10 mM NaCl. (b) Bar graphs showing time constants obtained by global exponential fitting of the transient absorption data for WT under 0.1, 1, and 10 mM Na⁺ conditions. Visible transient absorption measurement of WT in 1 mM NaCl condition is shown in Figure S2a.


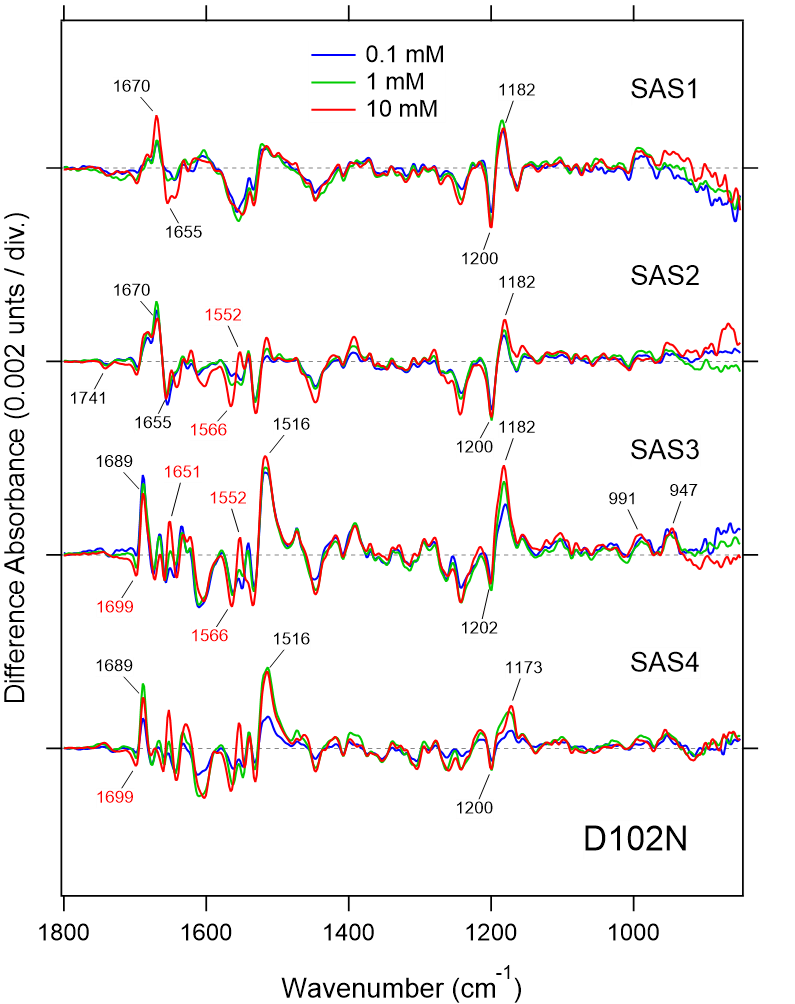


**Figure S6.** SAS1–4 spectra obtained from time-resolved FTIR spectroscopy of the D102N mutant films prepared from proteoliposome suspensions in buffers containing 0.1, 1, or 10 mM NaCl. Spectra recorded under 0.1, 1, and 10 mM Na⁺ conditions are shown in blue, green, and red, respectively.


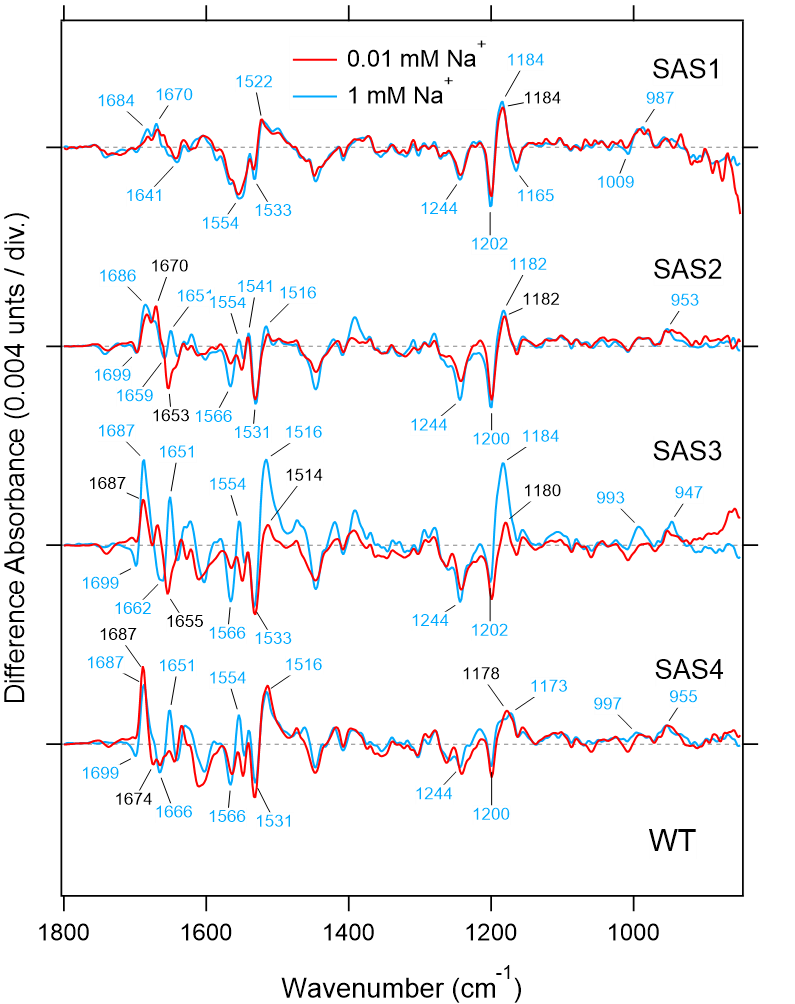


**Figure S7.** SAS1–4 spectra obtained from time-resolved FTIR spectroscopy of WT films prepared from proteoliposome suspensions in buffers containing 0.01 and 1 mM NaCl. Spectra recorded under 0.01 and 1 mM Na⁺ conditions are shown in red and light blue lines, respectively.


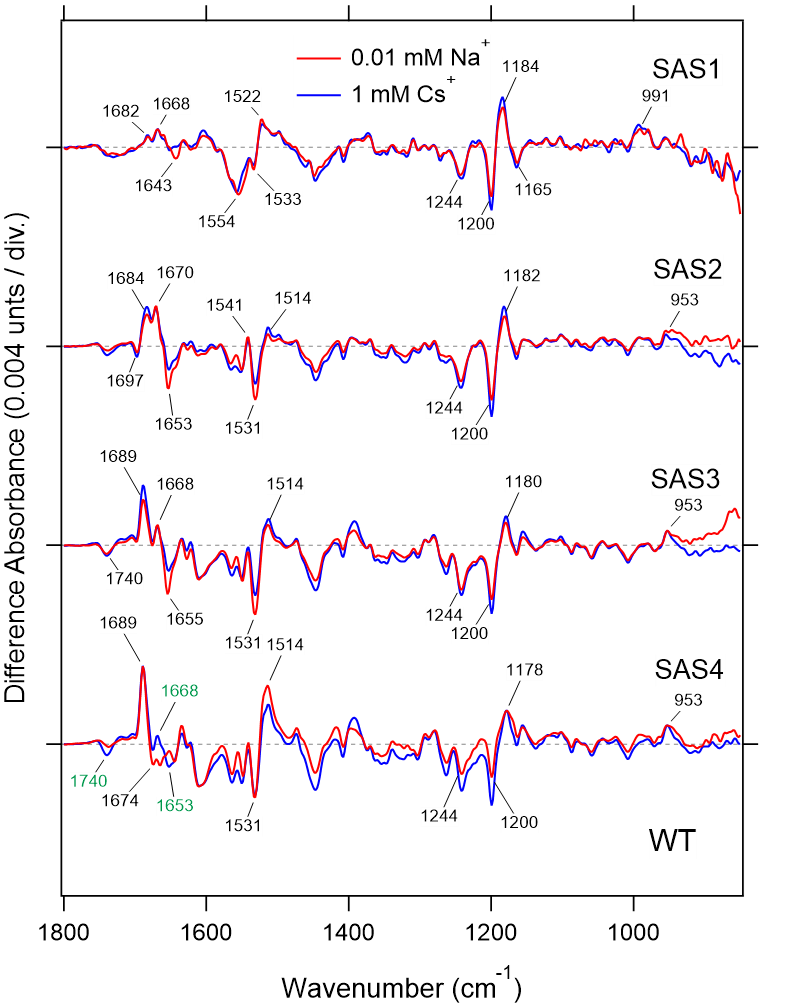


**Figure S8.** SAS1–4 spectra obtained from time-resolved FTIR spectroscopy of WT films prepared from proteoliposome suspensions in buffers containing 0.01 mM NaCl or 1 mM CsCl. Spectra recorded under 0.01 mM Na^+^ and 1 mM Cs⁺ conditions are shown in red and blue lines, respectively.

|  | τ_K_ (μs) | τ_L/M_ (μs) | τ_O1_ (ms) | τ_O2_ (ms) |
| --- | --- | --- | --- | --- |
| WT (H_2_O) | 97.5 | 740 | 2.08 | 8.02 |
| WT (D_2_O) | 97.6 | 731 | 2.51 | 9.77 |
| N112D | 182 | 1490 | 19.3 | 168 |
| ^13^C uniform | 114 | 646 | 2.14 | 8.38 |
| ^15^N uniform | 100 | 804 | 1.75 | 8.07 |
| C_15_-D retinal | 134 | 678 | 2.30 | 8.57 |
| C_14_-D retinal | 96.4 | 963 | 2.64 | 10.8 |
| C_12_-D retinal | 117 | 733 | 3.11 | 9.68 |
| C_11_-D retinal | 98.1 | 1110 | 1.92 | 9.58 |
| C_10_-D retinal | 102 | 857 | 1.75 | 6.65 |
| C_8_-D retinal | 94.8 | 1030 | 2.94 | 10.8 |
| C_7_-D retinal | 69.5 | 866 | 2.73 | 11.5 |

**Table S1.** Time constants obtained from time-resolved FTIR measurements of WT KR2 hydrated with H_2_O or D_2_O, as well as from stable isotope–labeled samples, including systematically deuterated retinal analogs and the N112D mutant.

|  |  | τ_K_ (μs) | τ_L/M_ (μs) | τ_O1_ (ms) | τ_O2_ (ms) |
| --- | --- | --- | --- | --- | --- |
| WT | 0.1 mM Na^+^ | 93.5 | 924 | 2.73 | 13.7 |
|  | 1 mM Na^+^ | 97.5 | 740 | 2.08 | 8.02 |
|  | 10 mM Na^+^ | 134 | 864 | 1.44 | 6.08 |
| D102N | 0.1 mM Na^+^ | 59.2 | 1950 | 4.15 | 10.0 |
|  | 1 mM Na^+^ | 53.5 | 820 | 1.95 | 9.06 |
|  | 10 mM Na^+^ | 101 | 588 | 2.26 | 37.2 |

**Table S2.** Time constants obtained from time-resolved FTIR spectroscopy of WT KR2 and the D102N mutant at different Na⁺ concentrations (0.1, 1, and 10 mM)
